# WAMD: From Pathophysiology to Therapeutic Treatments

**DOI:** 10.3390/biomedicines10081996

**Published:** 2022-08-17

**Authors:** Feliciana Menna, Alessandro Meduri, Stefano Lupo, Enzo Maria Vingolo

**Affiliations:** 1 Department of Ophthalmology, Kantonsspital Aarau (KSA), Tellstrasse 25, 5001 Aarau, Switzerland; 2 Department of Biomedical and Dental Science and of Morphological and Functional Images, University of Messina, 98122 Messina, Italy; 3 Department of Medical-Surgical Sciences and Biotechnologies, U.O.C. Ophthalmology, Sapienza University of Rome, Via Firenze 1, 04019 Terracina, Italy

Age-related macular degeneration (AMD) is referred to as the leading cause of irreversible visual loss in developed countries, with a profound effect on the quality of life. The neovascular form of AMD is characterized by the formation of subretinal choroidal neovascularization, leading to sudden and severe visual loss [1]. Aging of the eye is accompanied by the buildup of uncleared cellular debris that originates from the retinal pigment epithelium (RPE) and accumulates where the RPE interfaces with Bruch’s membrane and the neurosensory retina. These deposits, known as drusen, are typically the first ophthalmoscopic sign of AMD, appearing before visual function is appreciably affected. Drusen are composite structures, primarily consisting of lipids as well as proteins and carbohydrates that can be visualized as small white or yellowish deposits on the macula [2]. Drusen deposition in Bruch’s membrane concomitant with other structural and biochemical changes associated with AMD pathogenesis (including persistent activation of the complement cascade and inflammation) lead to thickening and decreased permeability of the membrane [3]. This obstructs both nutrient transport to the retina and waste exchange to the choroid and is accompanied by the thinning of the choroidal vasculature. These steps, combined with neurodegenerative changes within the photoreceptor–RPE complex, result in pigmentary abnormalities of the RPE, including hypo- or hyperpigmentation, in early or intermediate stages of disease [4].

The introduction of anti-VEGF intravitreal injections has opened a new therapeutic window in the management of wet AMD, thus efficiently blocking the pathophysiological process of AMD, with a restoration of retinal morphology and the maintenance of its function. Injections are considered safe, with few adverse reactions [5]. In the last few years, anti-VEGF injections have become the standard treatment for wet AMD, accounting for better results than previous treatments, such as photodynamic therapy (PDT) and laser photocoagulation [1].

Intravitreal injections of anti-VEGF agents have been recommended as a first-line treatment for neovascular AMD. However, persistent fluid or recurrent exudation still occurs despite standardized anti-VEGF therapy. Patients suffering from refractory or recurrent neovascular AMD may develop mechanisms of resistance to anti-VEGF therapy, which results in a diminished therapeutic effect [6]. Due to the highly heritable nature of AMD, it has been hypothesized that genetic factors may influence response to therapy for AMD and that the personalization of therapy may result in better outcomes. Genetic markers are independent of disease duration and therefore may explain treatment outcome variability [7].

In the last decade, a large number of studies have investigated the associations of genetic polymorphisms with anti-VEGF treatment responses in nAMD. Recently, it was found that the severity of AMD macular lesions is associated with rs2285714 and rs2230199 polymorphisms. Patients with the minor allele of rs2285714 respond worse to anti-angiogenic therapy [8]. The following observations can be made from the analysis of the literature. First, genetic predisposition contributes to the resistance to anti-VEGF therapy. Secondly, the finding of SNPs associated with the response to anti-VEGF therapy is important for the early identification of nAMD patients who may benefit from alternative therapies such as drug switching.

Optical coherence tomography (OCT) angiography (OCTA) represents an extremely powerful non-invasive approach to the detailed analysis of MNV secondary to AMD [9]. Quantitative OCTA parameters, in the first instance, MNV vessel tortuosity (VT), have recently been proposed to differentiate between clinically different MNV subgroups [10]. This quantitatively based categorization of the MNV lesions depended on the perfusion features of the MNV and its clinical activity and proved to be less influenced by the type of MNV. Thanks to OCTA it was shown that two different MNV subforms can be identified: low-vessel tortuosity MNV, which is more exudative at the baseline but less damaging to the outer retinal structures, and high-vessel tortuosity MNV, which is less exudative at the baseline but tends to lead to atrophic changes and functional deterioration [11]. In other recent studies, it was qualitatively described and quantified morphological differences among treatment-naïve types 1, 2 and 3 MNV [12]. OCTA imaging allows the detailed analysis of the pathologic vasculature of MNV, in contrast to FA, where the differentiation of vascular structures is particularly impeded by leakage.

Retinal pigment epithelium detachments (PEDs), which represent the separation between the basal lamina of the retinal pigment epithelium (RPE) and the inner collagenous layers of Bruch’s membrane, are considered to be prominent features of AMD. Three main types of PED have been described: serous, drusenoid and fibrovascular detachments. The exact pathophysiology of PEDs is still unknown. This seems to be related to the accumulation of fluid and debris due to the malfunction of a degenerated Bruch’s membrane [13]. Since there seems to be a correlation between their morphological structure and their evolution to exudative AMD, the interest in PEDs’ pathogenesis and their prognostic value has increased. In a recent study by Lupidi et al. [14], the authors reported that PEDs’ SD-OCT appearance might be considered a phenotypic prognostic factor for quiescent MNV. Particularly, they found the growth in PEDs’ greatest linear diameter to be associated with the maintenance of a quiescent phenotype, whereas the growth in PEDs’ maximal height was associated more frequently with the development of an exudative phenotype. Recently, a new humanized single-chain variable fragment was developed and named Brolucizumab (also known as RTH258). By reducing its dimensions compared to the previous anti-VEGF drugs, this new agent promises to better penetrate the targeted retinal tissues, to reduce immunogenicity and to provide a longer durability [15]. As an early response to a single intravitreal Brolucizumab injection in a recent study, PEDs showed a significant reduction in their maximal heights, whereas no significant difference was found in terms of horizontal maximal diameter, flow area and BCVA [16].

The best treatment for AMD depends on several factors, including the stage of the disease. Either way, in all disease stages, the elimination of risk factors—for instance, smoking cessation—is appropriate [17]. Dietary supplements for AMD have been widely discussed in the literature. However, the supplementation was found to have only a small effect on the intermediate stage, and no effect was found in the early or late stages of the disease [18]. The early detection of AMD may thus help motivate the patient to change lifestyle habits that promote the progression of the disease.

At present, there are few intravitreal injections of the VEGF antagonist available and used for the treatment of neovascular AMD, such as Pegaptanib sodium (Macugen^®^), Bevacizumab (Avastin^®^), Ranibizumab (Lucentis^®^), Aflibercept (Eylea^®^), Brolucizumab-Dbll (Beovu^®^) and Faricimab-Svoa (Vabysmo^®^).

Thus, an ideal biomarker to serve as a predictive response to the treatments has not yet been found. There is great controversy between reports due to the fact that there are no clear criteria to treat neovascular AMD and patients with variability in the duration of treatment, number of administered doses, samples and nationality are thus compared in the studies. In addition, there are different criteria to define the response to the treatment. Recently, new methods based on the combination of transcriptomic with genomic and proteomic methods have been proposed to identify patients with a poor response to anti-VEGF treatment and to establish better patient-specific treatment plans [19].

Despite all the efforts that the scientific community have directed towards neovascular AMD research, the treatment options currently available remain suboptimal. Intravitreal injections, the current standard of care, are invasive and expensive procedures that require frequent patient monitoring. Nonetheless, drug research and development is an ever-evolving field and several more therapies, such as complement pathway modulators, gene therapy and cell implants, are in the pipeline, promising future breakthroughs.
